# Effect of load sequence interaction on bond-wire lifetime due to power cycling

**DOI:** 10.1038/s41598-021-84976-2

**Published:** 2021-03-10

**Authors:** Zoubir Khatir, Son-Ha Tran, Ali Ibrahim, Richard Lallemand, Nicolas Degrenne

**Affiliations:** 1 SATIE Laboratory, Gustave Eiffel University, 25 Allee des Marronniers, 78000 Versailles, France; 2 Mitsubishi Electric R&D Centre Europe (MERCE), 1, Allee de Beaulieu CS 10806, 35708 Rennes, France

**Keywords:** Electrical and electronic engineering, Mechanical engineering

## Abstract

Experimental investigations on the effects of load sequence on degradations of bond-wire contacts of Insulated Gate Bipolar Transistors power modules are reported in this paper. Both the junction temperature swing ($\Delta T_{j}$) and the heating duration ($t_{ON}$) are investigated. First, power cycling tests with single conditions (in $\Delta T_{j}$ and $t_{ON}$), are performed in order to serve as test references. Then, combined power cycling tests with two-level stress conditions have been done sequentially. These tests are carried-out in the two sequences: low stress/high stress (LH) and high stress/low stress (HL) for both $\Delta T_{j}$ and $t_{ON}$. The tests conducted show that a sequencing in $\Delta T_{j}$ regardless of the direction “high-low” or “low–high” leads to an acceleration of degradations and so, to shorter lifetimes. This is more pronounced when the difference between the stress levels is large. With regard to the heating duration ($t_{ON}$), the effect seems insignificant. However, it is necessary to confirm the effect of this last parameter by additional tests.

## Introduction

In field operations, power semiconductor devices undergo several and complex stress conditions (thermal, vibration, humidity,…) with variable load conditions during their useful life. However, the commonly used procedure to estimate the remaining useful lifetime of power modules is based on accelerated life tests combined with the use of stress counting methods^1,2^. Accelerated aging tests applied to insulated-gate-bipolar-transistor power modules (IGBTs) have revealed two main failure mechanisms: the bond-wire degradations (heel-crack or lift-off) and solder delamination (die or DBC attach)^3–5^. Nevertheless, such aging tests are performed under single and fixed load conditions^3,4,6,7^ and lead to empirical lifetime models^8–10^. Concerning the stress counting, the most popular method is the rainflow method that is widely used for on-line estimation of the remaining useful lifetime^11,12^. This method is used to transform a complex mission profile with random stress amplitudes into a set of classified simple stress instances. It was originally proposed by Endo and Matsuishi^13^ for which one of the most used algorithms is described in^14^. Its use assumes that the Palmgren–Miner rule should be satisfied, i.e. the damage accumulation can be considered linear^15^ and therefore there is no interaction between load levels, neither in amplitude nor in duration or in sequencing. This considerably simplifies the remaining useful lifetime assessment of power modules. In many cases, this method gave satisfactory results^1,16,17^.

In the case of solder joints, an underestimation or overestimation of the damage accumulation has been reported for simultaneous application of thermal and vibration loads^18,19^. Similar results have been observed with the sequential loading of vibration and thermal stresses that have led to a deviation of the linear accumulation of damage in solder joints^20–22^.

Concerning the cumulative damage behavior of bond-wires in power electronic modules, one can cite Rajaguru et al.^23^ who compared linear and nonlinear methods for wire-bond damage accumulation under various thermal load amplitudes. The results showed that almost all nonlinear methods give similar lifetime results to the linear one. Nevertheless, the study was based solely on simulations without experimental validation.

In^24^, Zeng et al. have performed combined power cycling tests in junction temperature swing ($\Delta T_{j}$), minimum junction temperature ($T_{jmin}$) and load pulse duration ($t_{ON}$). They have shown that linear cumulative theory is rather confirmed for bondwire failures. Nevertheless, several stress parameters are modified at the same time in the combined tests and this does not allow to clearly study the effect of a combination of single stress parameter at a time. Furthermore, the conditions under which the hypothesis of linear cumulative damage might be questioned are not clearly established for the thermal fatigue of bonding wires.

The aim of this paper is to provide experimental results and discussion about the effects of loading sequence in aluminum bondwire damage accumulation of IGBT power modules during power cycling tests. Both the load amplitude, i.e. the junction temperature swing ($\Delta T_{j}$), and the load period, i.e. the heating duration ($t_{ON}$), will be investigated. The effects of these parameters will be methodically studied by modifying only one stress parameter at a time.

In a first step, power cycling tests with single conditions (in $\Delta T_{j}$ and $t_{ON}$), are performed in order to serve as test references. Then, power cycling tests with two-level stress conditions have been done sequentially. These tests will be carried-out in the two sequences: low stress/high stress (LH) and high stress/low stress (HL) for both $\Delta T_{j}$ and $t_{ON}$.

In order to avoid triggering several failure mechanisms, i.e. solder delamination and bondwire contact degradation, a well-suited power module with only bond-wire degradations has been chosen.

## Experimental aging tests

### Devices under test

**Figure 1:**
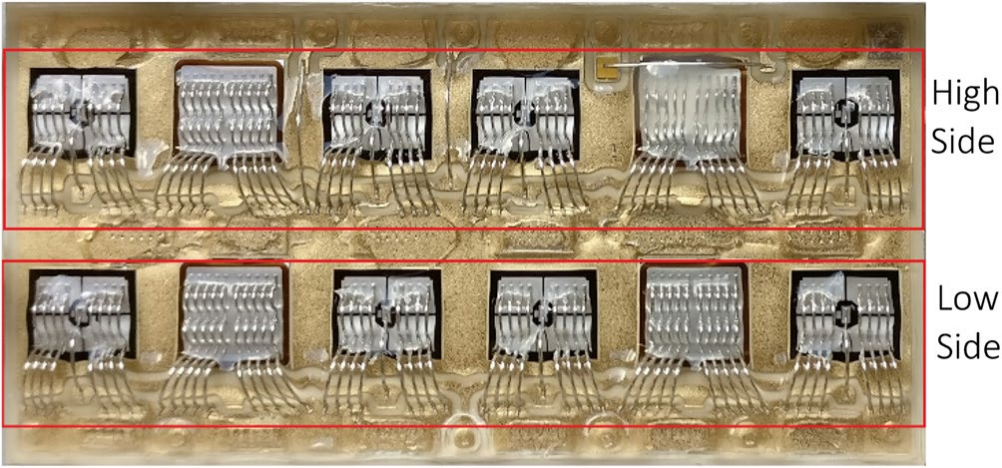
DBC substrate of IGBT power module.

The tested modules are commercially available SKIM63 (1200 V–300 A). These devices are six-pack modules with three individual phase legs on separated DBC substrates and without base plate. A photo of a substrate is given in Fig. 1. These power modules were chosen for their base plate-free and high-reliable die attach in order to concentrate the degradations only in the topside interconnections. The chips are all silver-sintered making the die-attach stronger than those using classical solders. Hence, the observed degradations concern only the chip metallization and the bond-wire contacts.

The power cycling is carried out only on the central leg of tested modules which constitutes 2 DUTs respectively high side and low side switches as visible in Fig. 1. Each DUT includes 4 IGBTs and 2 freewheel diodes on the DBC. In addition, the DBC is mounted on a heatsink by pressure contacts and pre-applied thermal grease without lead-frame.

### General methodology

A DC power cycling bench have been used for these tests. Three power modules, that is to say six DUTs, were tested for each test condition. The ageing indicators are collector-emitter voltage drop ($V_{ce}$) and junction-to-case thermal resistance ($R_{thjc}$). During the aging, the power cycling is interrupted and these parameters are always measured in the same conditions, in static regime. To this end, the power cycling test is automatically and regularly interrupted in order to perform the characterizations of ageing indicators. The junction temperature ($T_{j}$) is measured by a thermo-sensitive electrical parameter (TSEP), i.e. the collector-emitter voltage $V_{ce}$ (at $V_{ge}$ = 15 V, $I_{c}$ = 50 mA). The case temperature, below each IGBT chip, is measured by thermocouples for evaluating junction-to-case ($R_{thjc}$) thermal resistance. Failure criteria for stopping the tests use the above damage indicators: the drop voltage $V_{ce}$ (when it reaches 5% increase) or the junction-to-case thermal resistance $R_{thjc}$ (when it reaches 20% increase). In addition, in the test protocol, the $V_{ce}$ measurements are corrected from a possible increase in temperature which would be due to the increase in thermal resistance. This in such a way that the increase in $V_{ce}$ is only due to the topside interconnections. Full details on test methodology are given in^10^.

**Figure 2:**
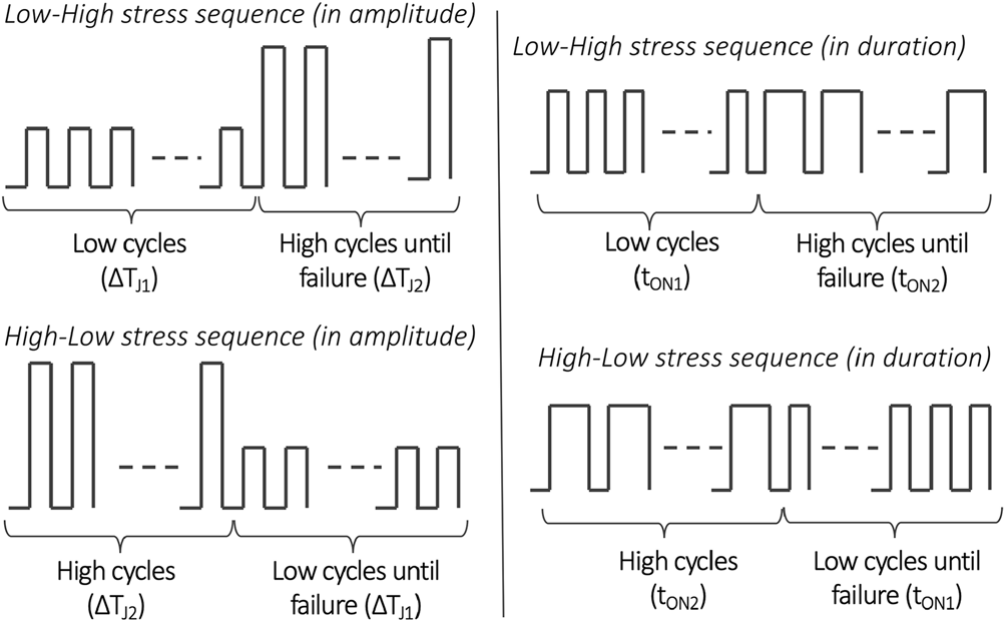
Load sequences in stress amplitude (left) and stress duration (right).

Sequential stress tests have been done with two stress levels as shown in Fig. 2:

- Tests in stress amplitude (in $\Delta T_{j}$) in both low–high (LH) and high-low (HL) sequences;
- Tests in stress duration ($t_{ON}$) also in low–high (or short-long) and high-low (or long-short) sequences.

Table 1 gives the two groups of tests performed: single level (or reference) tests from test#1 to test#4 and combined stress tests from test#5 to test#10 with respect to the reference tests. It also summarises the junction temperature swing ($\Delta T_{j}$), the minimum junction temperature ($T_{jmin}$), the heating time ($t_{ON}$), the cooling time ($t_{OFF}$) and the load current ($I_{L}$). Generally, for a single stress level, the degradation levels in % increase of $V_{ce}$ which correspond to the half-life ($N_{f}$/2) of the DUTs are in the range [1% to 2%], depending on the test conditions. As criterium to stop the first stress level before going to the second one is that DUTs undergo significant stress and degradations during both step levels. This leads to consider the degradation level (% in $V_{ce}$ increase) in the range 1% to 2%, instead of number of cycles. Then, the second stress level is applied until end of life (EOL). All tests are performed with a $T_{jmin}$ of 55 °C.

**Table 1:** Reference tests (single level) and sequenced tests (two-step levels) performed.

Test #	ΔT_j_ (°C)	T_jmin_ (°C)	t_ON_ (s)	t_OFF_ (s)	I_L_ (A)
**Single level (reference tests)**					
1	110	55	3	6	260
2	90	55	3	6	240
3	70	55	3	6	210
4	110	55	20	40	227
**Two-step levels (combined tests)**					
5	Test#1 until 1.5% increase in Vce then test#2 until EOL				
6	Test#2 until 1.1%… then test#1…				
7	Test#1 until 1.5%… then test#3…				
8	Test#3 until 1.7%… then test#1…				
9	Test#1 until 1.5%… then test#4…				
10	Test#4 until 1.3%… then test#1…				

**Figure 3:**
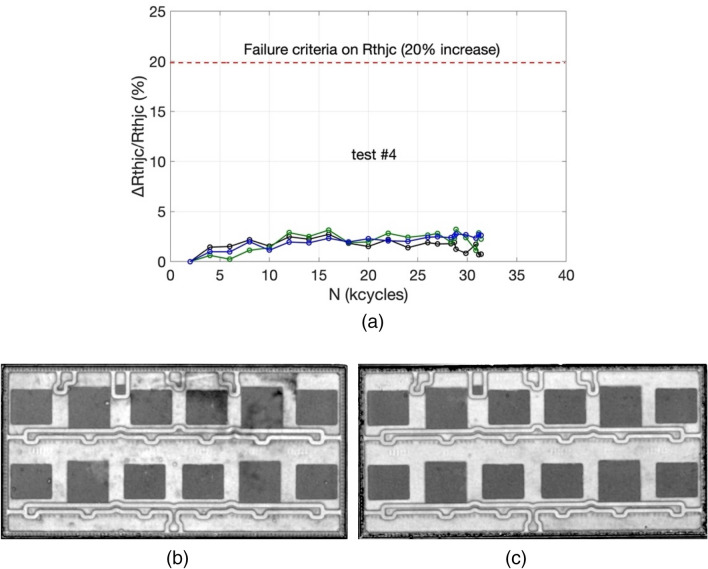
(**a**) Junction-to-case thermal resistance relative variation (test#4). Test conditions: T_jmin_ = 55 °C, ∆T_j_ = 110 °C, t_ON_/t_OFF_ = 20 s/40 s. SAM images of a DBC substrate respectively before (**b**) and after (**c**) test#4.

It has been verified that all tests (in Table 1) have led to the same damage and failure mode, i.e. at the top-side interconnection, and no degradation has been detected at the die attach. This is inferred from the measured thermal resistance that show unsignificant variations, indicating that there has been no delamination occurring in the structures. As illustration, the relative variation of junction-to-case thermal resistance ($R_{thjc}$) for the most impacting test condition on this parameter, i.e. with the longest heating time (t_ON_ = 20 s) is shown in Fig. 3a. The small $R_{thjc}$ variations is due to measurement difficulties when using thermal grease and contact pressure and is considered not significant enough. This was confirmed by scanning acoustic microscopy (SAM) analyses performed before and after ageing. The SAM images of a module before and after ageing (test#4) are shown in Fig. 3b demonstrating no degradation in the die-attach layers.

## Experimental results

### Results on $\Delta T_{j}$ ($t_{ON}$ = 3 s)

In Table 2, the results of the reference tests (single stress level) are given, where $N_{{f_{ref} }}$ is the number of cycles to failure for each DUT and $\overline{N}_{{f_{ref} }}$ is the mean value for each test. Since 3 modules are tested for each stress condition with 2 DUTs per module (high and low sides), it could have up to 6 lifetime results. Nevertheless, it is not possible to continue aging with a single DUT of the same module (top side or bottom side) because the thermal coupling becomes different and will alter the results. Thus, when the first of the 2 DUTs in each module reaches the failure criterion, the aging test is stopped and we only have 3 lifetime results per test condition. However, when the aging of the 2 DUTs of the same module is very close, we can still obtain 2 aging results. This is why we have 3 and sometimes 4 results reported in Table 3.

**Table 2:** Lifetime results of reference tests.

Test#	*N*_*fref*_ (kcycles)				$\overline{N}_{fref} \left( {{\text{kcycles}}} \right)$
1	56.9	56.5	56.9	64.5	58.7
2	134.1	148.3	160.0	162.8	151.3
3	822.3	900.5	899.0	–	973.9
4	29.3	33.7	33.4	–	32.1

**Table 3:** Lifetime results of sequenced tests.

Test#	$N_{1}^{seq} \;({\text{kcycles}})$	$N_{2}^{seq} \;({\text{kcycles}})$				$\overline{N}_{2}^{seq} \;({\text{kcycles}})$
5	40.0	53.4	61.1	63.0	63.4	60.2
6	56.6	26.0	32.2	33.3	34.7	31.6
7	40.0	307.9	327.3	343.1	–	326.1
8	400.0	8.0	9.0	9.0	12.0	9.8
9	40.0	5.3	6.3	6.6	–	6.0
10	20.1	26.6	31.1	36.9	37.1	32.9

Table 3 relates to the sequenced tests (step-change in levels). For a given test, all the samples undergo $N_{1}^{seq}$ cycles of the first level of stress, then a variable number $N_{2}^{seq}$ until the end of life (EOL). Here too, the number of results for these tests is 3 or 4.

For clarity in all experimental graphs below, the colored curves are related to reference tests: red curves to $\Delta T_{j}$ = 110 °C, the green curves to $\Delta T_{j}$ = 90 °C, the blue curves to $\Delta T_{j}$ = 70 °C, and finally the black curves are related to sequenced tests. All tests shown in this section are given for heating time $t_{ON}$ = 3 s.

**Figure 4:**
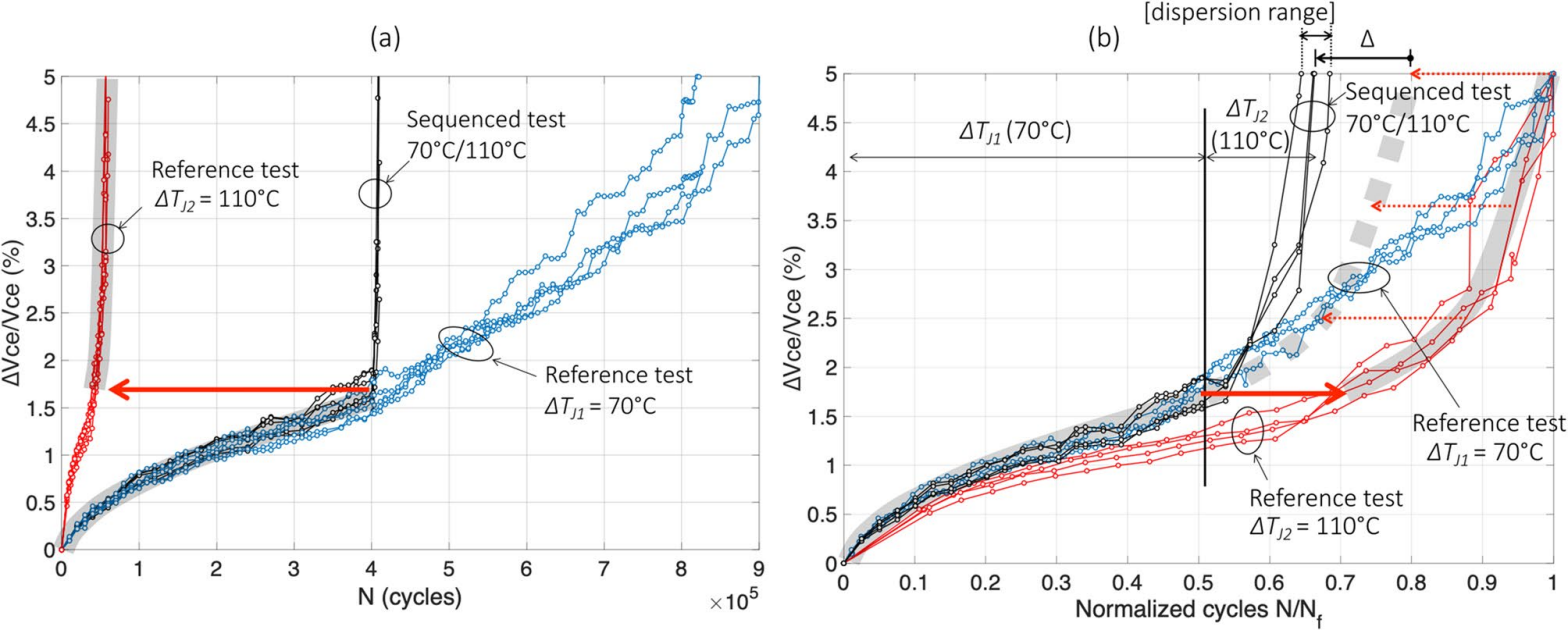
The sequenced test 70 °C/110 °C (test#8), normal scale in cycles (**a**) and normalized in ${\text{N}}_{{\text{f}}}$ (**b**).

Figure 4 reports the results of test#8 for the evolution of the relative variation in $V_{ce}$ with the cycles. In Fig. 4a, one can see that the sequenced-test curves (in black) begin with $\Delta T_{j1}$ = 70 °C and follow, as expected, the reference test at same $\Delta T_{j}$ (blue curves). Then, the test was switched to the second stress level ($\Delta T_{j2}$ = 110 °C) when $V_{ce}$ reaches 1.7% increase, and the black curves increase more sharply like the reference test at $\Delta T_{j}$ = 110 °C. In this figure, however, a faster increase in $V_{ce}$ in the sequenced test during the second period of aging than in the reference is observed.

The same results are presented in Fig. 4b in normalized scale. Each reference test (colored curves) is normalized with its own $N_{{f_{ref} }}$ value (see Table 2). Thus, each reference curve naturally ends at unity value. Concerning the combined test (black curves), each stress level is normalized by the corresponding reference mean value $\overline{N}_{{f_{ref} }}$ value (see Table 2). This last graph, allows to more clearly observe that, in this low–high stress sequence, in the second stress level, i.e. after 1.7% increase, aging is faster (black curves) than in the reference test (red curves).

In the following, we use the following notations:

- $N_{1}^{seq} /\overline{N}_{{f_{ref1} }}$ is the cycle number in the first step of sequenced test normalized with the $\overline{N}_{f}$ value of the corresponding reference test;
- $\overline{N}_{2}^{seq} /\overline{N}_{{f_{ref2} }}$ is the cycle number in the second step of sequenced test normalized with the $\overline{N}_{f}$ value of the corresponding reference test;
- $N_{1}^{ref} /\overline{N}_{{f_{ref1} }}$ is the equivalent normalized cycle number of reference test at first level for ∆$V_{ce}$/$V_{ce}$ < 1.7% (mean blue curve, or first part of grey trend curve); The value of 1.7% is for the test case 8 (see Table 1).
- $N_{2}^{ref} /\overline{N}_{{f_{ref2} }}$ is the equivalent normalized cycle number of reference test at second level for ∆$V_{ce}$/$V_{ce}$ ≥ 1.7% for the test#8 case (mean red curve, or second part of grey trend curve).

So, the difference:

1 $$ \Delta = \left( {\frac{{N_{1}^{seq} }}{{\overline{N}_{{f_{ref1} }} }} + \frac{{\overline{N}_{2}^{seq} }}{{\overline{N}_{{f_{ref2} }} }}} \right) - \left( {\frac{{N_{1}^{ref} }}{{\overline{N}_{{f_{ref1} }} }} + \frac{{N_{2}^{ref} }}{{\overline{N}_{{f_{ref2} }} }}} \right) $$

is a measure for the deviation from the Miner's rule for the sequenced tests. The greater the difference from zero, the stronger the effect of the stress sequence:

- If ∆ = 0, the effect of the stress sequencing is negligible as well as the deviation with Miner's rule;
- If ∆ > 0, the effect of the stress sequence exists, and the second stress level will slow the aging process (compared to what it would have been without effect of the sequence);
- If ∆ < 0 : the effect of the stress sequence exists, and the second stress level will accelerate the aging process (compared to what it would have been without effect of the sequence);

If tests are made rigorously, the first part of sequenced test is very similar to the corresponding reference test and thus: $N_{1}^{seq} /\overline{N}_{{f_{ref1} }}$ ≈ $N_{1}^{ref} /\overline{N}_{{f_{ref1} }}$, so that:

2 $$ \Delta \approx \overline{N}_{2}^{seq} /\overline{N}_{{f_{ref2} }} - N_{2}^{ref} /\overline{N}_{{f_{ref2} }} . $$

**Figure 5:**
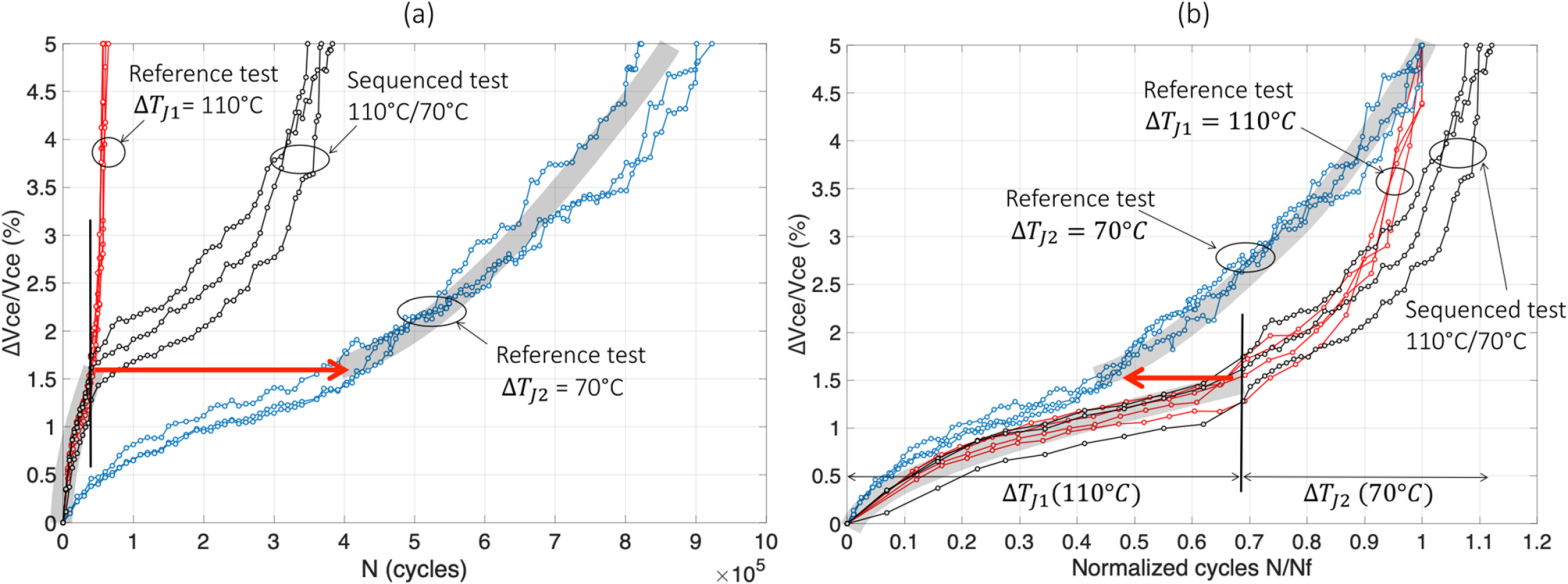
The sequenced test 110 °C/70 °C (test#7), normal scale in cycles (**a**) and normalized in ${\text{N}}_{{\text{f}}}$ (**b**).

For example, in the case of test#8, one can read in Fig. 5b: $N_{1}^{seq} /\overline{N}_{{f_{ref1} }}$ ≈ 0.5 (see mean black curve until stress switch) and $\overline{N}_{2}^{seq} /\overline{N}_{{f_{ref2} }}$≈ 0.17 (see mean black curve from stress switch). One can also read : $N_{1}^{ref} /\overline{N}_{{f_{ref1} }}$ ≈ 0.5 (mean blue curve, or grey trend curve, for ∆$V_{ce}$/$V_{ce}$ < 1.7%), and $N_{2}^{ref} /\overline{N}_{{f_{ref2} }}$ ≈ 0.3 (mean red curve, or grey trend curve, for ∆$V_{ce}$/$V_{ce}$ ≥ 1.7%) :

$$ \Delta_{{70\;^{ \circ } {\text{C}} \to 110\;^{ \circ } {\text{C}}}} \approx - 0.13. $$

In other words, for test#8, if the Miner's rule were satisfied the second part should look like the red curves (given by the trend of the thick grey line) from 1.7% in Vce. In this case, the number of normalized cycles of this second part would have been $N_{2}^{ref} /\overline{N}_{{f_{ref2} }}$ ≈ 0.3, i.e. from 0.5 (beginning of the thick dotted grey line) to 0.8 (end of the thick dotted grey line). For the passage from 1st stress level to the 2nd, only the amount of degradation reached counts. The deviation from the Miner’s rule is given by ∆ in the left direction (∆ < 0), see Fig. 4b.

This result indicates that in this low–high stress sequence, during the second stress level, aging is faster (black curves) than in the reference test (red curves). The dispersion in the ∆ value is in the range [− 0.15; − 0.12].

The results of test#7 are reported in Fig. 5. In Fig. 5a, the sequenced curves (in black) begin with $\Delta T_{j1}$ = 110 °C and follow, as expected, the reference test at same $\Delta T_{j}$ (red curves). Then, when ∆$V_{ce}$/$V_{ce}$ reaches 1.5% increase, the second stress level ($\Delta T_{j2}$ = 70 °C) is applied and the black curve increases more sharply than the corresponding reference test (blue curves).

In Fig. 5b, the same results are presented in normalized scale for both reference tests. It can be observed that, in this high-low stress sequence, in the second stress level, i.e. after 1.5% increase, aging is also faster (black curves) than in the reference test (blue curves). In this case, $N_{1}^{seq} /\overline{N}_{{f_{ref1} }}$≈ 0.69, $\overline{N}_{2}^{seq} /\overline{N}_{{f_{ref2} }}$≈ 0.41, $N_{1}^{ref} /\overline{N}_{{f_{ref1} }}$≈ 0.69 (mean red curve, or grey trend curve, for ∆$V_{ce}$/$V_{ce}$ < 1.5%), and $N_{2}^{ref} /\overline{N}_{{f_{ref2} }}$≈ 0.55 (mean blue curve, or grey trend curve, for ∆$V_{ce}$/$V_{ce}$ ≥ 1.5%). The difference due to the sequencing effect is approximately:

$$ \Delta_{{110\;^{ \circ } {\text{C}} \to 70\;^{ \circ } {\text{C}}}} \approx - 0.14 $$

with a dispersion rather low, in the range [− 0.15; − 0.13]. This value is quite the same as that for the reverse sequence: $\Delta_{{70\;^{ \circ } {\text{C}} \to 110\;^{ \circ } {\text{C}}}}$ ≈ $\Delta_{{110\;^{ \circ } {\text{C}} \to 70\;^{ \circ } {\text{C}}}}$.

In summary, for these two-step tests, whatever the sequence “low–high” or “high-low”, having different levels of stress leads to an acceleration in aging during the second stress period. Both tests have led to a reduction in $\overline{N}_{2}^{seq} /\overline{N}_{{f_{ref2} }}$ of about 0.13.

**Figure 6:**
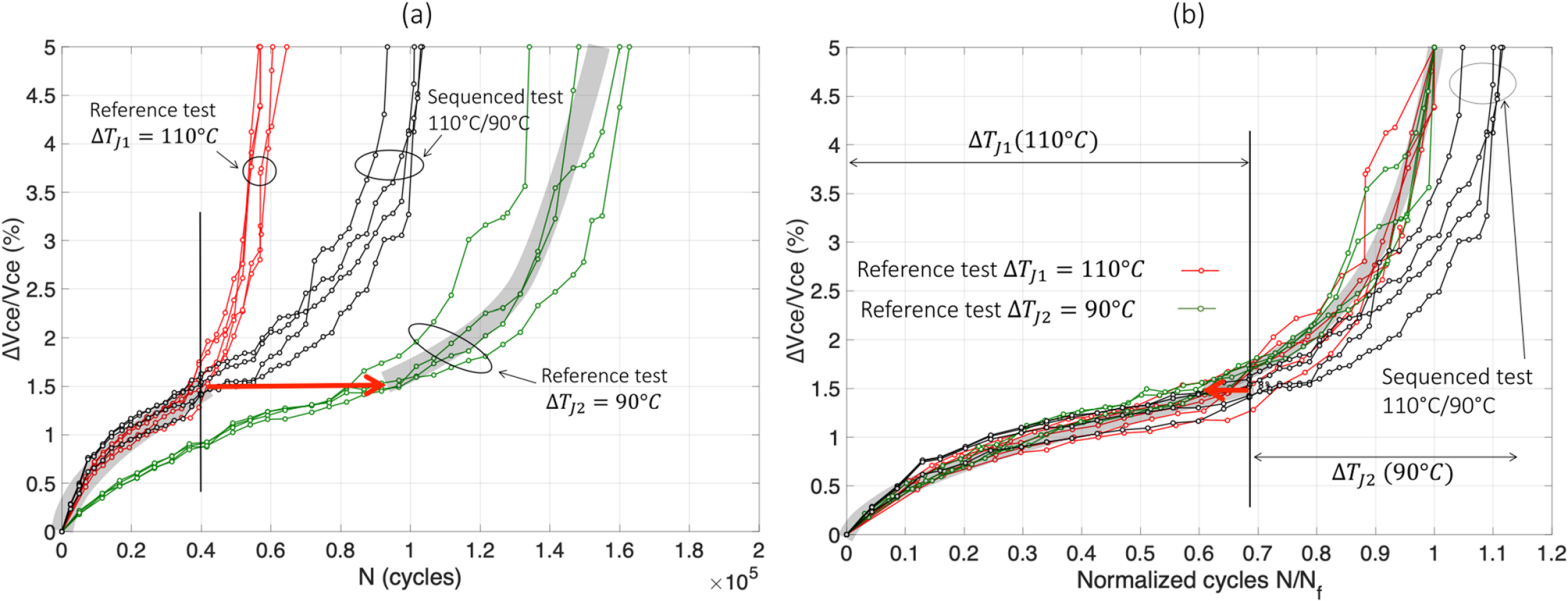
The sequenced test 110 °C/90 °C (test#5), normal scale in cycles (**a**) and normalized in ${\text{N}}_{{\text{f}}}$ (**b**).

The results of test#5 are reported in Fig. 6. The sequenced curves (in black), in Fig. 6a, start with $\Delta T_{j1}$ = 110 °C and follow, as expected, the corresponding reference test (red curves). Then, when ∆$V_{ce}$/$V_{ce}$ reaches 1.5% increase, the second stress level ($\Delta T_{j2}$ = 90 °C) is applied and the black curves increase almost as the reference test at same $\Delta T_{j}$ (green curves). In this figure, the shape and rate of rise of $V_{ce}$ increase seem to be the same as in the second part of aging.

In Fig. 6b, the same results are presented in normalized scale in $N_{f}$ for both reference tests. It can be observed that, in this stress sequence, in the second stress level, i.e. after 1.5% increase, aging is rather the same (black curves) as that in the reference test (green curves) taken into account the shift in red arrow.

In this case, $N_{1}^{seq} /\overline{N}_{{f_{ref1} }}$≈ 0.69, $\overline{N}_{2}^{seq} /\overline{N}_{{f_{ref2} }}$≈ 0.4, $N_{1}^{ref} /\overline{N}_{{f_{ref1} }}$ ≈ 0.69 (mean red curve, or grey trend curve, for ∆$V_{ce}$/$V_{ce}$ < 1.5%), and $N_{2}^{ref} /\overline{N}_{{f_{ref2} }}$≈ 0.4 (mean green curve, or grey trend curve, for ∆$V_{ce}$/$V_{ce}$ ≥ 1.5%). There is no effect due to this sequencing, the mean value is:

$$ \Delta_{{110\;^{ \circ } {\text{C}} \to 90\;^{ \circ } {\text{C}}}} \approx 0 $$

with a dispersion in the range [− 0.03; 0.03].

**Figure 7:**
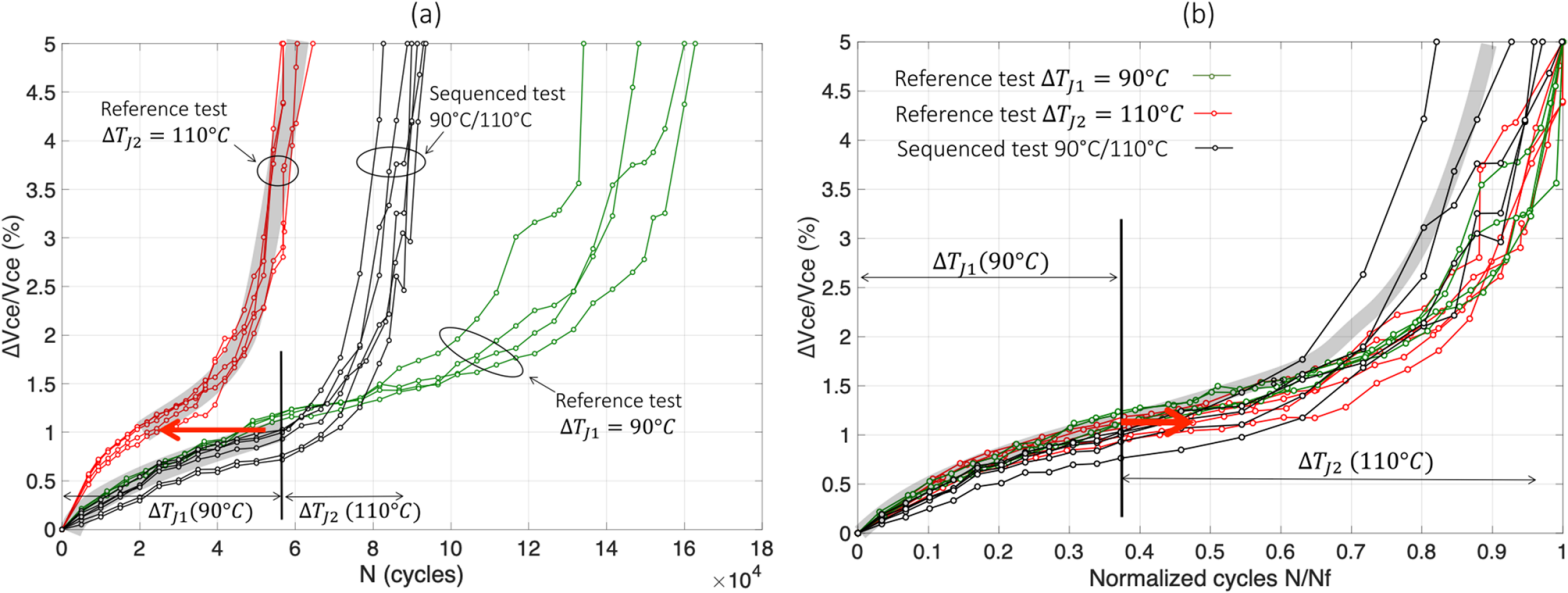
The sequenced test 90 °C/110 °C (test#6), normal scale in cycles (**a**) and normalized in ${\text{N}}_{{\text{f}}}$ (**b**).

The results of test#6 are reported in Fig. 7. The sequenced curves (in black) in Fig. 7a, start with $\Delta T_{j1}$ = 90 °C and follow with a slightly lower values, the reference test at same $\Delta T_{j}$ (green curves). Then, the test was switched to the second stress level ($\Delta T_{j2}$ = 110 °C) when $V_{ce}$ reaches 1.1% increase, and the black curve increases almost as the corresponding reference test at $\Delta T_{j}$ = 90 °C. The mean $V_{ce}$ increase, in the black curves, seems nevertheless slightly faster than in the red curves.

In Fig. 7b, the same results are presented in normalized scale for both reference tests. It can be observed that, in this low–high stress test, in the second stress level, i.e. after 1.1% increase, aging is slightly faster (mean black curves trend) than that in the corresponding reference test (mean red curves).

In this case, $N_{1}^{seq} /\overline{N}_{{f_{ref1} }}$≈ 0.37, $\overline{N}_{2}^{seq} /\overline{N}_{{f_{ref2} }}$≈ 0.46, $N_{1}^{ref} /\overline{N}_{{f_{ref1} }}$≈ 0.37 (mean green curve, or grey trend curve, for ∆$V_{ce}$/$V_{ce}$ < 1.1%), and $N_{2}^{ref} /\overline{N}_{{f_{ref2} }}$≈ 0.53 (mean red curve, or grey trend curve, for ∆$V_{ce}$/$V_{ce}$ ≥ 1.1%). The effect due to this sequencing is weak, in average it is:

$$ \Delta_{{90\;^{ \circ } {\text{C}} \to 110\;^{ \circ } {\text{C}}}} \approx - 0.05 $$

but with a dispersion rather large, in the range [− 0.08; 0.1]. Compared to tests #7 and #8, the analysis for tests #5 and #6 is difficult taking into account the large variations in aging results (dispersion) and the relatively low differences between stresses (90 °C and 110 °C in $\Delta T_{j}$) compared to the previous ones.

In summary, for the latter test, the stress sequence “high–low” does not seem to lead to a change in the rate of aging, while the “low–high” sequence has a small effect with a weak acceleration of aging during the second period of stress.

### Results on $t_{ON}$ ($\Delta T_{j}$ = 110 °C

For clarity in result presentation, in all graphs below, the colored curves are related to reference tests: pink curves are related to $t_{ON}$ = 3 s and the blue ones to $t_{ON}$ = 20 s. The black curves are related to sequenced tests. All tests shown in this sub-section are given for $\Delta T_{j}$ = 110 °C. So, the pink curves in the following graphs are the same as the red curves in the previous sub-section.

**Figure 8:**
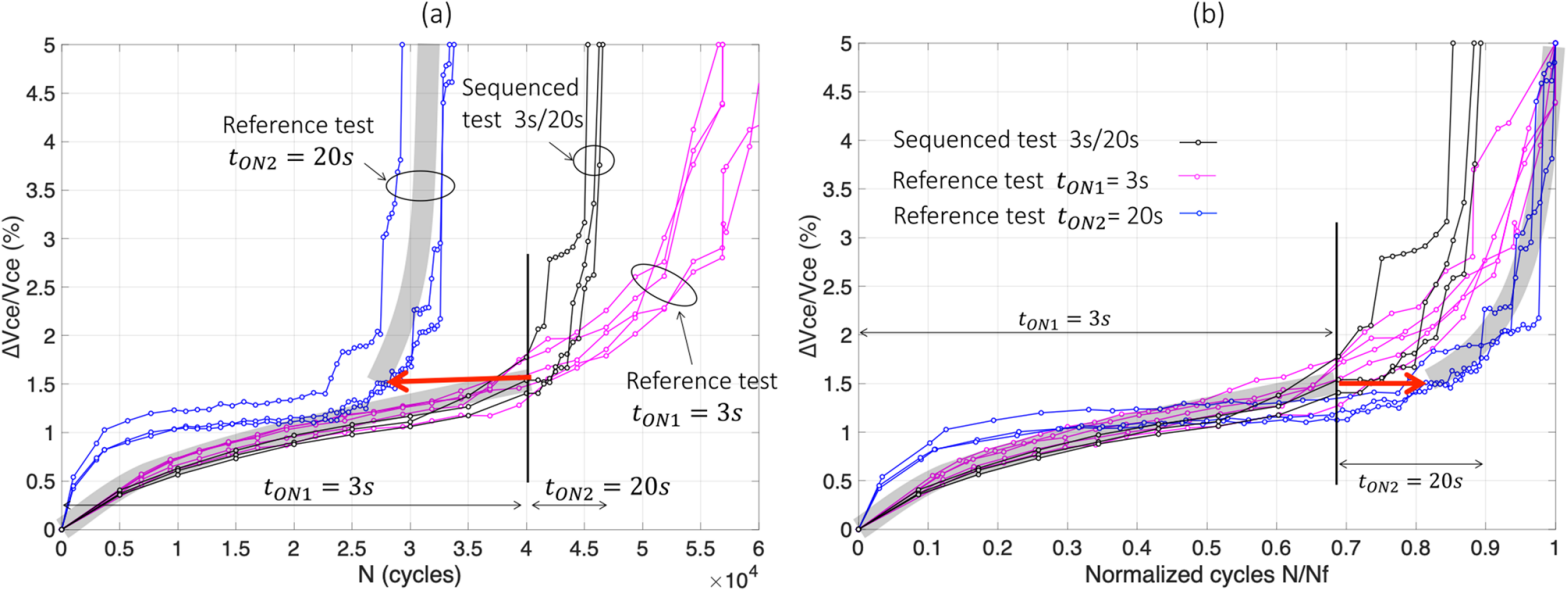
The sequenced test 3 s/20 s (test#9), normal scale in cycles (**a**) and normalized in ${\text{N}}_{{\text{f}}}$ (**b**).

The results of test#9 are reported in Fig. 8. It can be observed that in a first stage, in Fig. 8a, that he sequenced curves (in black), begin with $t_{ON}$ = 3 s and follows, as expected, the corresponding reference test at same $t_{ON}$ (pink curves). Then, when $V_{ce}$ reaches 1.5% increase, the second stress level ($t_{ON}$ = 20 s) is applied and the black curves increase like the corresponding reference test, at quite the same rate of rise.

In Fig. 8b, the same results are presented in normalized scale. In this case, $N_{1}^{seq} /\overline{N}_{{f_{ref1} }}$≈ 0.68, $\overline{N}_{2}^{seq} /\overline{N}_{{f_{ref2} }}$≈ 0.2, $N_{1}^{ref} /\overline{N}_{{f_{ref1} }}$ ≈ 0.69 (mean pink curve, or grey trend curve, for ∆$V_{ce}$/$V_{ce}$ < 1.5%), and $N_{2}^{ref} /\overline{N}_{{f_{ref2} }}$≈ 0.18 (mean blue curve, or grey trend curve, for ∆$V_{ce}$/$V_{ce}$ ≥ 1.5%). The difference due to the sequencing effect is tiny:

$$ \Delta_{{3\;{\text{s}} \to 20\;{\text{s}}}} \approx + 0.01 $$

with a dispersion centered in the range [-0.02; + 0.02].

**Figure 9:**
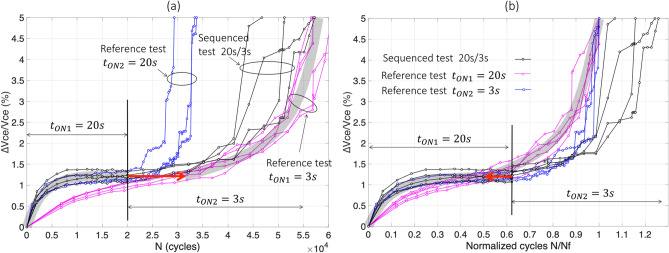
The sequenced test 20 s/3 s (test#10), normal scale in cycles (**a**) and normalized in ${\text{N}}_{{\text{f}}}$ (**b**).

Finally, the results of test#10 are reported in Fig. 9. In Fig. 9a, the sequenced curves (in black), begin with $$t_{ON} $$ = 20 s and follows, as expected, the corresponding reference test (blue curves). Then, when $$V_{ce}$$ reaches 1.3% increase (mean values), the second stress level ($$t_{ON}$$ = 3 s) is applied and the black curves increase like the corresponding reference test with a slightly slower average trend.

In Fig. 9b, the same results are presented in normalized scale where $N_{1}^{seq} /\overline{N}_{{f_{ref1} }}$≈ 0.62, $\overline{N}_{2}^{seq} /\overline{N}_{{f_{ref2} }}$≈ 0.56, $N_{1}^{ref} /\overline{N}_{{f_{ref1} }}$ ≈ 0.62 (mean blue curve, or grey tendancy curve, for ∆$V_{ce}$/$V_{ce} { }$ < 1.3%), and $N_{2}^{ref} /\overline{N}_{{f_{ref2} }}$≈ 0.5 (mean pink curve, or grey tendancy curve, for ∆$V_{ce}$/$V_{ce}$ ≥ 1.3%). The gap due to the sequencing effect is tiny:

$$ \Delta_{{20\;{\text{s}} \to 3\;{\text{s}}}} \approx + \;0.06 $$

with a dispersion in the range [− 0.05; + 0.1]. This shows that the double-stress sequencing leads to a slightly slower aging process during the second stress level compared to the corresponding reference. Nevertheless, the effect remains too small, given the dispersion of the measured data-points and it is difficult to draw a conclusion on these basis.

In summary, these two-test sequence in $t_{ON}$ lead to insignificant impact in reducing very slightly the aging process during the second stress period, whatever the sequence “low–high” or “high–low”. These values in ∆ are so small that confirmations should be performed with a much larger difference in stress levels.

Finally, the results obtained from the two-level test stress seem to indicate that only tests at significantly different $\Delta T_{j}$ levels (between 110 and 70 °C) allow the interaction effects to be observed. These systematically lead to an acceleration of aging. On the other hand, if the differences between the $\Delta T_{j}$ are low (for example between 110 and 90 °C), the effects are weak and the Miner’s rules^15^ seem to apply. Regarding the effects of the $t_{ON}$, here again they seem negligible and in line with Miner's rule premise.

Nevertheless, all these results were obtained in accelerated conditions, where the main degradations are due to visco-plastic strains. It is necessary to investigate how these results will be affected in normal conditions of use and especially, when degradations are mainly due to visco-elastic deformations. Furthermore, these tests were done to highlight the effects of two-level sequence on the lifetime. It will also be necessary to study the effects of a more complex sequencing, with multiple interlacing as in^24^. The study here implies that a more precise lifetime estimation can be obtained by considering the stress history not only for die-attach^25^, but also for wire-bonds.

## Conclusion

The results presented here concern only the bondwire contact degradation. The tests conducted seem to show that a sequencing in $\Delta T_{j}$, regardless of the direction “high–low” or “low–high” leads to an acceleration of degradations in the second phase of stress. This is more pronounced as the difference between the stress levels is large. For small differences between the two stress levels, the effect of sequencing is reduced or even annulled.

With regard to the heating duration ($t_{ON}$), the effect seems to be negligible. Nevertheless, given the weak interaction obtained between the stresses, these last results must be confirmed by additional tests by increasing the difference between the two levels of $t_{ON}$.

In addition, all these results were obtained in accelerated conditions, where the main degradations are due to visco-plastic strains. It remains to investigate how these results will be affected in more realistic conditions and especially, when degradations are mainly due to visco-elastic deformations, and/or in combination with visco-plastic strains.

More works are needed to understand the underlying physics and why this sequencing leads to an acceleration or reduction of the aging rate. Further work is also needed for more complex sequencing (finer interleaving). In addition, of great interest is to generate data for the sequences combining stresses that are dominantly in the elastic domain, as our current understanding indicates that it is there that the power modules experience the larger portion of their life.

Once the above questions have been answered, a proper physics-of-failure framework can be created that can address problems in the domain of remaining useful lifetime estimation.
